# Interactions of Symbiotic Partners Drive the Development of a Complex Biogeography in the Squid-Vibrio Symbiosis

**DOI:** 10.1128/mBio.00853-20

**Published:** 2020-05-26

**Authors:** Tara Essock-Burns, Clotilde Bongrand, William E. Goldman, Edward G. Ruby, Margaret J. McFall-Ngai

**Affiliations:** a Kewalo Marine Laboratory, Pacific Biosciences Research Center, University of Hawai‘i at Mānoa, Honolulu, Hawaii, USA; b Department of Microbiology and Immunology, University of North Carolina at Chapel Hill, Chapel Hill, North Carolina, USA; University of Connecticut

**Keywords:** symbiosis, biogeography, confocal microscopy, *Euprymna scolopes*, *Vibrio fischeri*, imaging, microbiogeography, microscopy

## Abstract

Microbes live in complex microniches within host tissues, but how symbiotic partners communicate to create such niches during development remains largely unexplored. Using confocal microscopy and symbiont genetics, we characterized the shaping of host microenvironments during light organ colonization of the squid Euprymna scolopes by the bacterium Vibrio fischeri. During embryogenesis, three pairs of invaginations form sequentially on the organ’s surface, producing pores that lead to interior compressed tubules at different stages of development. After hatching, these areas expand, allowing V. fischeri cells to enter and migrate ∼120 μm through three anatomically distinct regions before reaching blind-ended crypt spaces. A dynamic gatekeeper, or bottleneck, connects these crypts with the migration path. Once V. fischeri cells have entered the crypts, the bottlenecks narrow, and colonization by the symbiont population becomes spatially restricted. The actual timing of constriction and restriction varies with crypt maturity and with different V. fischeri strains. Subsequently, starting with the first dawn following colonization, the bottleneck controls a lifelong cycle of dawn-triggered expulsions of most of the symbionts into the environment and a subsequent regrowth in the crypts. Unlike other developmental phenotypes, bottleneck constriction is not induced by known microbe-associated molecular patterns (MAMPs) or by V. fischeri*-*produced bioluminescence, but it does require metabolically active symbionts. Further, while symbionts in the most mature crypts have a higher proportion of live cells and a greater likelihood of expulsion at dawn, they have a lower resistance to antibiotics. The overall dynamics of these distinct microenvironments reflect the complexity of the host-symbiont dialogue.

## Abstract (precis)

The complexity, inaccessibility, and time scales of initial colonization of most animal microbiomes present challenges for the characterization of how the bacterial symbionts influence the form and function of tissues in the minutes to hours following the initial interaction of the partners. Here, we use the naturally occurring binary squid-vibrio association to explore this phenomenon. Imaging of the spatiotemporal landscape of this symbiosis during its onset provides a window into the impact of differences in both host-tissue maturation and symbiont strain phenotypes on the establishment of a dynamically stable symbiotic system. These data provide evidence that the symbionts shape the host-tissue landscape and that tissue maturation impacts the influence of strain-level differences on the daily rhythms of the symbiosis, the competitiveness for colonization, and antibiotic sensitivity.

## INTRODUCTION

Bacterial symbionts occur in reproducible biogeographic patterns along the tissues of their animal hosts (1–5). For example, the biophysical and biochemical nature of tissue microenvironments fosters colonization by distinct microbial communities along the length of the mammalian gut and, at any given location, at various distances from the gut epithelial surface to its lumen (6–9). Several experimental studies with vertebrate systems have demonstrated that embryogenesis provides the host with a tissue architecture and biochemistry that define the niche space for initial colonization with specific members of the microbiota (10–13). However, because the microbiota of vertebrates is complex and largely inaccessible to fine-scale manipulation (14, 15), the natural progression of host-symbiont dialogue that results in the establishment and development of microscale biogeographic patterns along the symbiotic tissues remains poorly understood. Here, using the experimentally tractable, binary light organ symbiosis between the squid Euprymna scolopes and the luminous bacterium Vibrio fischeri, we define the dynamic tissue landscape that exists during colonization and how host-symbiont interactions shape the microenvironments to support symbiotic development.

**FIG 1:**
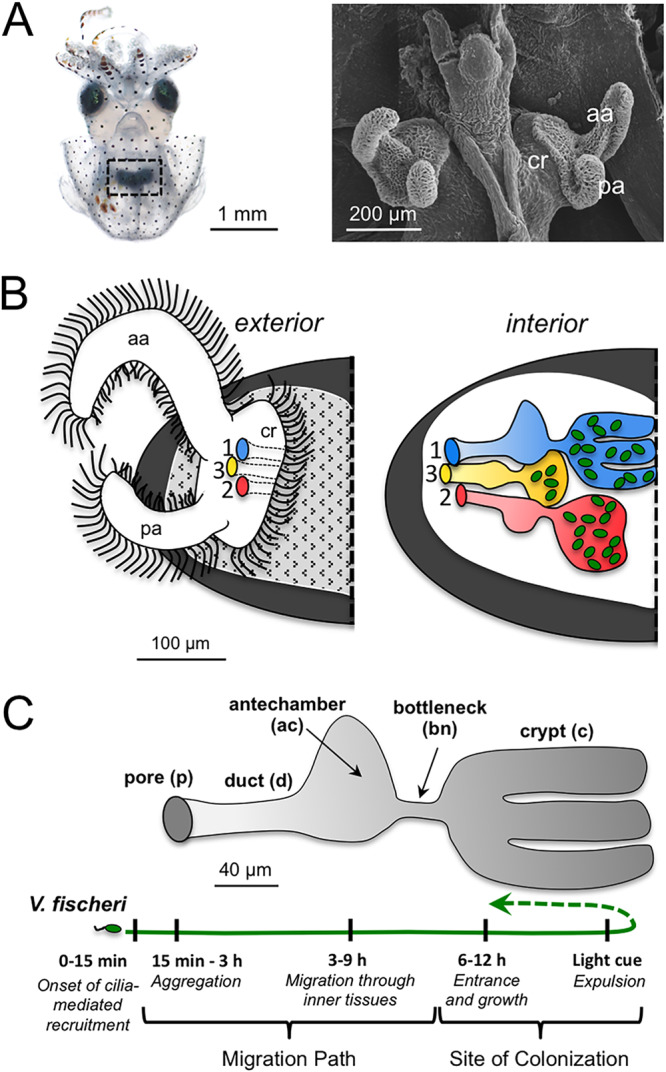
Microenvironments through which bacteria traverse to initiate colonization of host tissues. (A) A light micrograph of an E. scolopes hatchling shows the central location of the symbiotic light organ, which is embedded in the ink sac (black, dashed square) (left). A scanning electron micrograph of the bilobed light organ is shown (right). (B) A diagram of the exterior of one half of the light organ shows the position of the three pores on the surface (blue, red, and yellow) through which symbionts enter host tissues (left). An illustration of how each pore leads to three independent regions of various levels of maturity (1 > 2 > 3) and of their relative positions is shown at right. The distal regions are sites of V. fischeri (green) colonization. (C) A diagram of the five distinct microenvironments of each region and the timing of migration through the tissues. The first four are those through which V. fischeri cells traverse (migration path) to reach the crypt (far right), where the population resides (site of colonization) and from which the bacteria are vented each day at dawn. aa, anterior appendage; ac, antechamber; bn, bottleneck; c, crypt; cr, ciliated ridge; d, duct; p, pore; pa, posterior appendage.

Each generation, the squid host acquires its symbiont from the environment (Fig. 1A and B) (16). During embryogenesis, elements of the nascent organ are developed. Three invaginations on each lateral face of the developing light organ form sequentially to create microenvironments of various degrees of maturity that will become the routes of colonization of the crypts, which are epithelium-lined pockets deep within host tissues (Fig. 1B) (16). At hatching, each invaginated region is poised to transform into five anatomically and biochemically distinct microenvironments (Fig. 1C) (17). These regions comprise the migration path of environmental V. fischeri from the organ surface to the crypts, where symbiont populations reside throughout the life of the host (Fig. 1B and C). Specifically, for each invagination, from most superficial to deepest, the five regions include the following: (i) the pore, into which V. fischeri cells are recruited from ambient seawater, (ii) a narrow duct, (iii) a more expansive antechamber, (iv) a bottleneck, and (v) a crypt, where the symbionts grow and luminesce (Fig. 1C). The migration path from the pore to the crypt entrance is a biochemically harsh environment containing antimicrobials that prevent nonspecific bacteria from colonizing and deter V. fischeri cells from lingering (18, 19). Of the three crypts on either side of the organ, the first to develop (crypt 1) is the largest (75% of the total volume) and most mature at hatching, whereas the third (crypt 3) is the smallest (5% of the volume) and least mature, with crypt 2 as intermediate (16). Although these three crypts vary in degree of maturity, all can be colonized: on average, one V. fischeri cell enters a crypt and proliferates to a density that induces luminescence (20).

Upon colonization by the environmentally acquired V. fischeri, symbiosis-induced development of the light organ ensues (21). The resulting morphogenetic program begins within hours of colonization and unfolds across tens of micrometers in within hours to days; this short time frame allows for fine-scale spatiotemporal resolution of host-symbiont communication. Crypt colonization induces changes in the epithelial cells, including cell swelling, with an associated transformation from columnar to cuboidal, and an increase in microvillar density (22–24). The most exaggerated epithelial changes correlate with the gradient of crypt maturation from crypt 1 to crypt 3. Superficial light organ tissues are also affected by colonization of the crypts, where the ciliated field that mediates symbiont recruitment regresses over the first 4 days of symbiosis (25, 26). These symbiont-induced changes are reflected in transcriptional responses in the light organ (27–29), and even remote organs, such as the eyes and gills, are transcriptionally affected (28).

In addition to these early morphogenetic events, the light organ begins a daily rhythm in the first day following hatching. With the first dawn and throughout the life of the host thereafter, the cue of environmental light triggers the host to expel most of its symbiont population into the surrounding seawater (Fig. 1C). During this diel venting, 90 to 95% of the symbionts move back through the initial migration path and are released through the pore (30). Within hours of venting, the symbiont population remaining in the crypts has regrown (31) so that the animal is ready to use V. fischeri luminescence in its nocturnal behavior.

Taken together, this system offers spatiotemporal features that provide an opportunity to visualize, with high resolution, how a symbiotic system is initiated and stabilized. Whereas symbiont-induced changes have been studied in detail in the superficial and crypt epithelia, the role of symbionts in shaping their migratory path has been poorly defined. The results presented here reveal the following: (i) that both host-autonomous and symbiont-induced postembryonic development work in concert to create the landscape of the symbiotic association and (ii) that critical interactions between host and symbiont cells both restrict the symbionts to the crypt spaces during the day and facilitate their release with the dawn light cue. Applying here the rich platform of symbiont genetics, strain diversity, and biochemical determinates of host developmental phenotypes, this study enriches our understanding of the dynamics of host-symbiont interactions as they develop in a natural system.

## RESULTS

### Variation in tissue maturity across the light organ influences early symbiosis.

**FIG 2:**
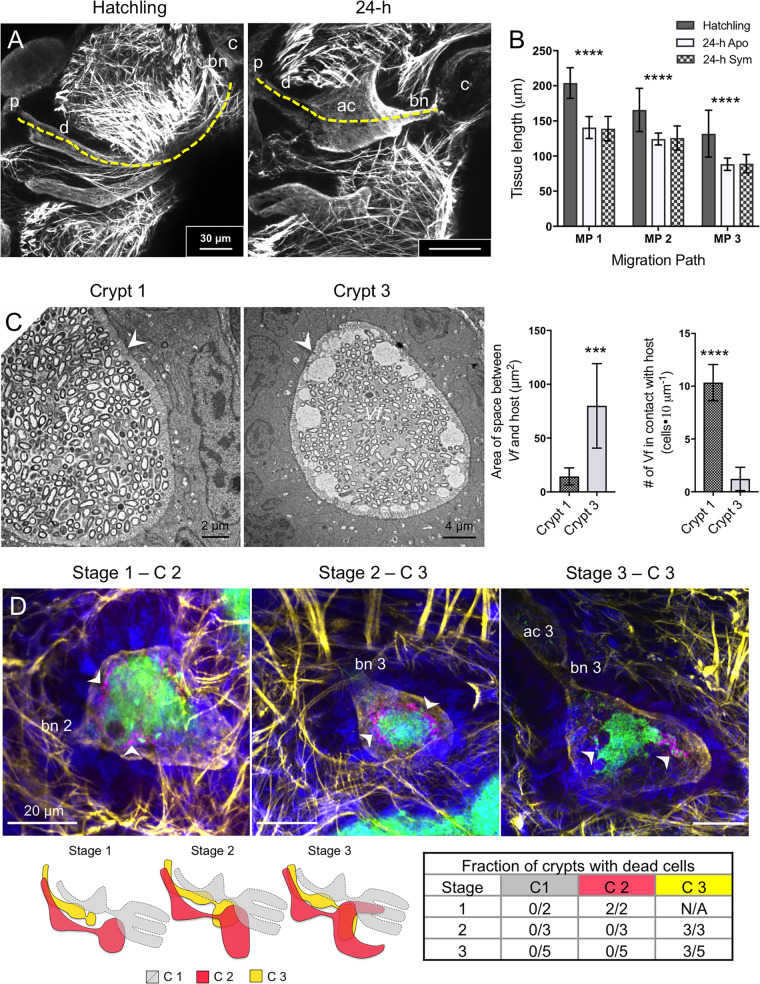
Early biophysical relationships that influence patterns of symbiosis onset. (A) Confocal micrographs show the migration path at hatching compared to that of a 24-h animal (symbiotic shown here). Projections of compressed stacks of a single channel show cytoskeletal actin and microvilli (phalloidin). The dashed, yellow line shows the two-dimensional estimate of length measured from the pore to the end of the bottleneck for each migration path. Bar, 30 μm. (B) The length of each path at hatching and in 24-h aposymbiotic or symbiotic animals was compared using a two-way ANOVA for each developmental stage (*F*_2, 142_ = 96.74; ****, *P < *0.0001). For each migration path (MP), the length of each migration path for the hatched, aposymbiotic, and symbiotic states, respectively, were as follows: for MP1, *n* = 19, 16, and 18; for MP2, *n* = 17, 16, and 20; for MP3, *n* = 16, 12, and 17. (C) Bacterial proximity to host tissue as a function of crypt maturation. TEM micrographs (left) of symbiotic crypts show the proximity of V. fischeri cells to host microvilli in crypt 1 and in crypt 3. Bar, 2 μm. Graphs at right show the area of the space between symbionts and host microvilli and the number of cells in direct contact with host microvilli, as indicated, in 24-h symbiotic animals (*n* = 3 biological replicates; 9 images per crypt). For each animal, three two-dimensional micrographs, taken at different tissue depths, were analyzed for each crypt type using an unpaired *t* test for distance (*T* = 4.924, df = 16, *P* = 0.0002) and cells (*T* = 13.46, df = 16, *P* < 0.0001). Values that are significantly different are indicated as follows: ***, *P* < 0.001; ****, *P* < 0.0001. (D) Distribution of dead bacteria in crypts. Confocal micrographs of animals at 24-h colonized by green fluorescent protein-labeled ES114 stained with an indicator of dead cells (magenta) are shown. Below, stages reflect variation of crypt maturation at hatching (left). The table (right) shows the prevalence of dead cells in crypts (relative to the total crypts). Blue, host nuclei stained with TOPRO-3; yellow, actin, stained with phalloidin, showing the terminal web of the epithelial layer lining the microenvironments. ci, cilia; mv, microvilli; *Vf*, Vibrio fischeri.

Following hatching, the migration paths leading to any crypt (Fig. 1C) shortened by an average of 24 to 33%, regardless of the presence of V. fischeri or other environmental bacteria (Fig. 2A and B; see Fig. S1A in the supplemental material). Also at hatching, whereas crypt 1 expanded within minutes (Fig. S1B), the less mature crypts 2 and 3 did not change in volume or shape.

10.1128/mBio.00853-20.1 FIG S1 Expansion of the microenvironments at hatching. (A) Representative confocal micrographs of each migration path (1 to 3) for hatchling, 24-h apo- and symbiotic tissues. Dashed, yellow line shows the length of tissue, from the pore (p) to the end of the bottleneck (bn) (quantified in Fig. 2B). Bar, 30 μm. (B) Confocal micrograph of a single optical section showing the difference between the collapsed crypt 1 morphology (left) and the expanded (right), a change that can occur with 2 min (F-actin labeling, phalloidin, magenta; host nuclei, TOPRO-3, blue). Arrowhead, bottleneck at crypt entrance; white line, outline of the terminal web of crypt epithelium. Bar, 15 μm. Download FIG S1, TIF file, 14.1 MB. Copyright © 2020 Essock-Burns et al. 2020 Essock-Burns et al. This content is distributed under the terms of the Creative Commons Attribution 4.0 International license.

We characterized by confocal and transmission electron microscopy (TEM) the host-symbiont interfaces along the migration path during initial colonization to gain insight into the potential nature of host interactions and to understand parallels to ciliated and microvillous symbiotic epithelia in other systems. The data provided evidence that whereas dense cilia were observed along epithelia of the migration path, they were not present in the crypts (Fig. S2 and S3). Further, the microvilli along the microenvironments leading to the crypts were longer and more uniform in their organization than those along the crypt epithelia (Fig. S2 and S3). In addition, unlike the crypt epithelium, which effaces at the time of symbiont expulsion at dawn and reorganizes over the hours following dawn (22, 23), the microvilli of the other regions were unchanged over the day in all migration paths. Thus, the bottlenecks define a separation in microvillar and ciliary characteristics between the epithelial cells of the migration path and those of the crypts (Fig. S3). In addition, symbiont cells were slightly more dense in crypt 1 (average of 7 cells per 10 μm^2^) than in crypt 3 [average of 5 cells per 10 μm^2^; *t*(16) = 2.129, *P* = 0.049]. Last, interaction of V. fischeri cells with the host cell surfaces varied with developmental maturity of the crypts; symbiont cells in the most mature crypt (crypt 1) were in direct contact with the microvilli, whereas those of the less mature crypts were not (Fig. 2C).

10.1128/mBio.00853-20.2 FIG S2 Variation in cilia and microvilli in different regions of the migration path. Transmission electron micrographs show cilia and microvilli in the duct, antechamber, and bottleneck along the terminal web of the epithelial layer (white arrowhead). Cilia are shown in cross-section (arrows), and microvilli (lighter gray) surround each cilium. ac, antechamber; bn, bottleneck; C1, crypt 1; d, duct; mv, microvilli; p, pore; r, cilia rootlets; tj, tight junction. Bar, 2 μm. Download FIG S2, TIF file, 14.1 MB. Copyright © 2020 Essock-Burns et al. 2020 Essock-Burns et al. This content is distributed under the terms of the Creative Commons Attribution 4.0 International license.

10.1128/mBio.00853-20.3 FIG S3 Apical surfaces of epithelium vary along microenvironments. Representative confocal micrographs of apical epithelial surfaces of each microenvironment correspond to the boxed regions in the drawing. Stacks of single channels show the microvilli and cytoskeletal actin stained with phalloidin (top row), and cilia are labeled with anti-acetyl-α-tubulin (bottom row). Bar, 20 μm. Download FIG S3, TIF file, 14.1 MB. Copyright © 2020 Essock-Burns et al. 2020 Essock-Burns et al. This content is distributed under the terms of the Creative Commons Attribution 4.0 International license.

We also assessed the viability of bacterial cells within the tissue as a function of crypt maturity. Whereas crypt 1 shows little developmental variation at hatching, the less mature crypts 2 and 3 have a wider range of maturation states (Fig. 2D) (17). This level of maturity is reflected in the viability of the symbiont cells within the crypts. Using a dead-cell indicator stain, we evaluated symbiont survival at 24 h postinoculation and regrowth (i.e., after the first venting). The majority of V. fischeri cells located in the crypts were alive. When dead cells were observed, they were most often in crypt 3, usually at the edge of the mass of live cells; the least mature stages of crypt development (i.e., stages 1 and 2) were most likely to contain dead symbionts (Fig. 2D). After venting, both live and dead cells were often observed in duct 1 near the pore (Movie S1); given the rarity of dead cells within crypt 1 itself, those observed in the migration path likely died after expulsion from the crypt environment.

10.1128/mBio.00853-20.8 MOVIE S1 Dead bacteria near the pore after the first vent. Video shows confocal optical slices using a dead-cell-indicator (red) on 24-h symbiotic animals. Green, live V. fischeri ES114 cells labeled with green fluorescent protein; blue, DNA labeled with TOPRO-3. The first slice is most superficial, just outside the pore and moves deeper into the pore/duct interface; host cells that comprise the pore are shown as large blue TOPRO-3-labeled features. Download Movie S1, MOV file, 4.1 MB. Copyright © 2020 Essock-Burns et al. 2020 Essock-Burns et al. This content is distributed under the terms of the Creative Commons Attribution 4.0 International license.

### The bottleneck region of the migration path is the most responsive to symbiosis, a phenotype that varies with the strain of colonizing V. fischeri.

**FIG 3:**
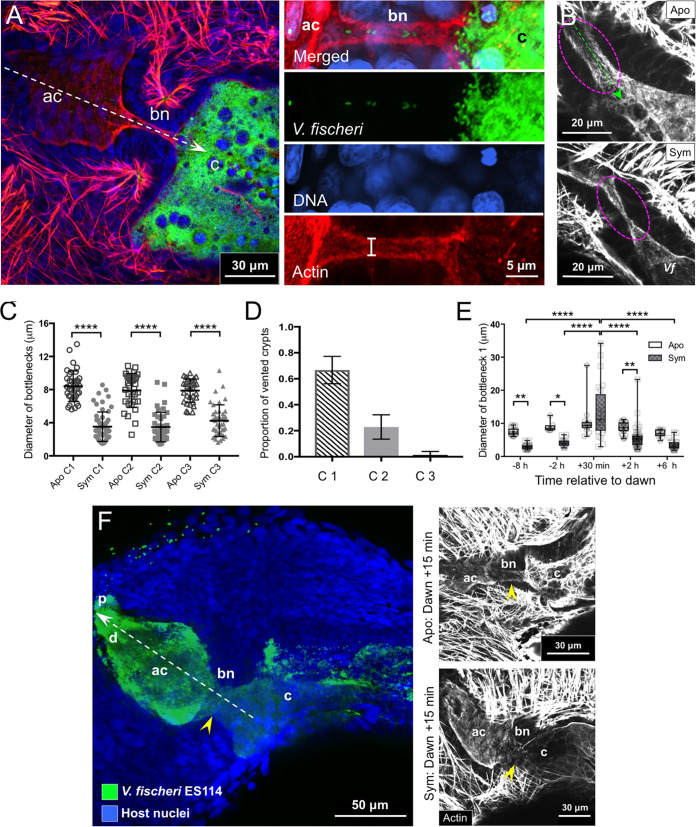
Bottleneck tissue through colonization and during venting. (A) Typical 24-h symbiotic tissues (left) exhibit a large population of green fluorescent protein-labeled V. fischeri cells in the crypts, which had moved through the migration path (white, arrow). A magnified confocal micrograph of a bottleneck is shown at right (top, merged image; green, green fluorescent protein label; blue, TOPRO-3 nuclear label; red, rhodamine-phalloidin actin label). White lines on the cross-section of the bottleneck in the actin channel show an example of the location measured for the bottleneck diameter. (B) Actin labeling shows aposymbiotic bottleneck tissue (magenta, dashed circle), which is open to the passage of symbionts (green, dashed arrow) (top). The constriction of the bottleneck in response to V. fischeri colonization of the crypt is also shown (bottom). (C) Diameter of each bottleneck type (1 to 3) is shown for aposymbiotic versus symbiotic crypts (C1 to C3). Each point represents a single animal, and each crypt was visualized to confirm colonization with green fluorescent protein-labeled V. fischeri. Groups were compared with a Kruskal-Wallis test and Dunn’s *post hoc* test (apo C1, *n* = 38; sym C1; *n* = 56, apo C2, *n* = 38; sym C2, *n* = 47; apo C3, *n* = 38; sym C3, *n* = 47) (*H* = 75.41, df = 131, *P < *0.0001). (D) The proportion of each symbiotic crypt type (C1 to C3) that vented across all animals was examined within 2 h of the dawn light cue (bars indicate the 95% confidence intervals of the mean; *n* = 77). Venting was measured as an open bottleneck (>6 μm) and with >20 green fluorescent protein-labeled V. fischeri cells in an antechamber associated with a colonized crypt. (E) The bottleneck 1 diameter over the day/night cycle by symbiotic state. Data were compared using a two-way ANOVA. The interaction of symbiotic state and time relative to dawn with bottleneck diameter was analyzed (*F*_4, 187_ = 5.989, *P = *0.0001), as well as symbiotic state (*P < *0.0001) and time relative to dawn (*P < *0.0001). For the aposymbiotic animals, at −8 h, *n* = 17; −2 h, *n* = 7; +30 min, *n* = 14; +2 h, *n* = 25, +6 h, *n* = 12. For the symbiotic state, at −8 h, *n* = 19; −2 h, *n* = 16; +30 min, *n* = 24; +2 h, *n* = 42; +6 h, *n* = 22). (F) Confocal micrograph shows the expulsion of V. fischeri (green) from the crypt back through (white, dashed arrow) a widened bottleneck (yellow arrowhead) and rest of the path (left). Actin staining (top right) shows the difference in the morphologies of a typical bottleneck (yellow arrowheads) between aposymbiotic animals and symbiotic animals (bottom right) at 15 min following the dawn light cue. ac, antechamber; apo, aposymbiotic; bn, bottleneck; c, crypt; d, duct; sym, symbiotic, *Vf*, Vibrio fischeri. Values that are significantly different are indicated as follows: ****, *P < *0.0001.

Because our characterization of the migration path revealed the bottlenecks to be the regions most responsive to early events of symbiosis (Fig. 1C and 3A), we sought to analyze their development and response to symbiosis more deeply. An earlier study showed that the bottlenecks narrow with colonization (Fig. 3B) (17), but the phenomenon was not defined fully. Data here showed that all three bottlenecks on each side of the organ constricted 46 to 58% only after V. fischeri had colonized the corresponding crypts (Fig. 3B and C), whereas the lengths did not change (Fig. S4), indicating that the narrowing is not due to a lateral stretching of the epithelium lining the bottlenecks.

10.1128/mBio.00853-20.4 FIG S4 Strain effects on bottleneck length. Bottleneck (BN1 to BN3) length in 24-h aposymbiotic and symbiotic tissues colonized by ES114, MB13B1, or MB13B2. Bottlenecks of animals colonized by MB13B1 were shorter than those of aposymbiotic animals, which correlates with their wider phenotype (Fig. 5). A Kruskal-Wallis test was used to compare treatments for bottleneck 1 (*H* = 21.71, df = 146, *P < *0.0001) and bottleneck 3 (*H* = 7.267, df = 129, *P = *0.0639), and Dunn’s multiple-comparison test was used to distinguish the groups in bottleneck 1. A one-way ANOVA and Tukey’s *post hoc* test was used to compare groups within bottleneck 2 (*F*_3, 128_ = 6.87, *P = *0.0003). Download FIG S4, TIF file, 14.1 MB. Copyright © 2020 Essock-Burns et al. 2020 Essock-Burns et al. This content is distributed under the terms of the Creative Commons Attribution 4.0 International license.

In addition to closing down with the onset of symbiosis, the bottleneck also facilitated venting. However, we found that not all crypts initially vent (66% of the time in crypt 1, 20% in crypt 2, and 1% in crypt 3) (Fig. 3D). Whereas the bottlenecks of crypts 2 and 3 close down with symbiosis, they do not open as fully as the bottleneck of crypt 1. Although the lack of full opening of the bottlenecks of crypts 2 and 3 may be involved in resistance to venting, other factors may contribute (e.g., amount of musculature around epithelia of the crypts). To explore the timing of this process, we examined crypt 1 before and after dawn at the time of the second presentation of the light cue, i.e., the second dawn postinoculation (Fig. 3E). The bottleneck of crypt 1 increased from a closed diameter of ∼4 μm at 2 h prior to dawn to a widened diameter of ∼13 μm within 30 min of dawn, and as a reflection of the venting phenotype, V. fischeri cells were observed in all portions of the migration path (Fig. 3F). The noise around this value reflects the known variation in timing of venting between individual animals in response to the dawn light cue. Within 2 h of dawn, the diameter of bottleneck 1 was much reduced (Fig. 3E). Taken together, these data show that this venting-associated expansion is transitory and that most of the day the bottlenecks of symbiotic crypts are closed, with the V. fischeri populations being confined to the crypts.

**FIG 4:**
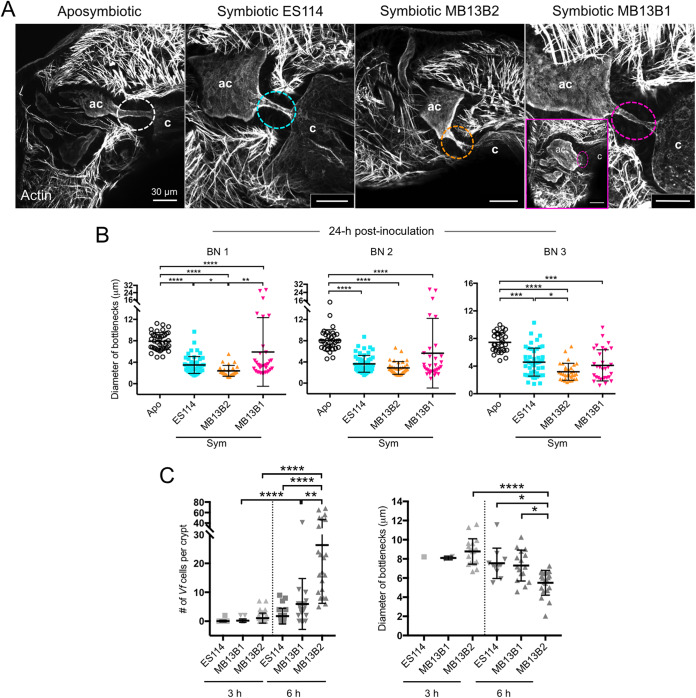
Effects of strain variation on the bottleneck at different stages of colonization. (A) Bottleneck (dashed circles) response was compared when crypts were colonized by three V. fischeri wild-type strains at 24 h postinoculation. Colonization by MB13B1 led to two phenotypes, one similar to that of the other two strains and a second in which the bottleneck was much wider and shorter (inset). Bar, 30 μm. (B) Quantification of strain variation in constriction of each bottleneck after 1 day of colonization. The colors of the dashed circles (in panel A) and data point symbols correspond to each strain, as indicated. A Kruskal-Wallis test was used to compare results among groups within a bottleneck type. For bottleneck type 1 (BN1), aposymbiotic, *n* = 41; ES114, *n* = 51; MB13B2, *n* = 26; MB13B1: *n* = 34 (*H* = 78.46, df = 151, *P < *0.0001). For bottleneck type 2, aposymbiotic, *n* = 33; ES114, *n* = 53; MB13B2, *n* = 35; MB13B1, *n* = 32, (*H* = 63.16, df = 152, *P < *0.0001). For bottleneck type 3, aposymbiotic, *n* = 30; ES114, *n* = 49; MB13B2, *n* = 32; MB13B1, *n* = 30 (*H* = 53.66, df = 139, *P < *0.0001). (C) Numbers of V. fischeri cells present in the crypts at 3 and 6 h postinoculation are shown (left). At right, diameters for all bottleneck types with V. fischeri present are shown for each time point (corresponding to left). A Kruskal-Wallis test and Dunn’s *post hoc* test were used. For crypts examined for cell number at 3 h: ES114, *n* = 42; MB13B1, *n* = 21; MB13B2, *n* = 48. For crypts examined for cell number at 6 h: ES114, *n* = 24; MB13B1, *n* = 20; MB13B2, *n* = 22 (*H* = 106.7, df = 176, *P < *0.0001). For crypts examined for bottleneck diameter at 3 h in MB13B2, *n* = 19. For crypts examined for bottleneck diameter at 6 h: ES114, *n* = 11; MB13B1, *n* = 16; MB13B2, *n* = 20 (*H* = 33.39, df = 65, *P < *0.0001). As at most 2 cells of ES114/MB13B1 are present in a crypt at 3 h in 1 to 2 cases (1/42 crypts for ES114 and 2/21 crypts for MB13B1), they were excluded from the statistical analysis. Values that are significantly different are indicated as follows: *, *P < *0.05; **, *P < *0.01; ***, *P < *0.001; ****, *P < *0.0001.

Using three diverse V. fischeri strains with distinct colonization strategies (32, 33), we asked whether strain-level differences occur in bottleneck constriction and in the timing of the response. When coinoculated under laboratory conditions, sharing strains, i.e., ES114 and MB13B1, typically cocolonize light organs, while a dominant strain, i.e., MB13B2, accesses the crypts more rapidly and rarely cocolonize the light organ (33). After colonization with a single strain for 24 h, all of the three strains typically induced the same level of bottleneck constriction although crypts colonized with the dominant strain MB13B2 had narrower bottlenecks than crypts colonized with the sharing strain ES114 (Fig. 4A and B). Occasionally (∼19% of the time at 24 h), the bottlenecks leading to a crypt colonized by the sharing strain MB13B1 were more open than those of aposymbiotic animals (Fig. 4A and B); however, they behave similarly to other strains in degree of bottleneck constriction by 48 h. Despite this expansive opening at 24 h, the MB13B1 symbionts remained confined to their crypts, suggesting that, together with the physical constriction, additional factors influence this symbiotic phenotype.

We also studied the variation in strain behavior during the initial induction of bottleneck constriction. Although we observed a few cells of the quickly colonizing dominant strain MB13B2 in the crypts after 3 h, all of the bottlenecks were still open (Fig. 4C). Very few crypts contained the two sharing strains of V. fischeri at 3 h postinoculation (Fig. 4C). As expected (33), all crypts contained MB13B2 cells after 6 h but typically fewer than 9 cells for ES114 and MB13B1, and the bottlenecks of the crypts colonized by MB13B2 had already begun to constrict (Fig. 4C and D). The results with different strains are consistent with the hypothesis that quickly colonizing strains, such as MB13B2, accelerate these changes as they do other symbiosis-induced modifications that occur during light organ morphogenesis (33, 34). Taken together, these data on strain variation provide evidence that the symbiotic phenotype of bottleneck constriction is an early host response to the presence of V. fischeri in crypts.

### Bottleneck response is independent of known symbiont morphogens but requires sustained interaction with the symbionts.

**FIG 5:**
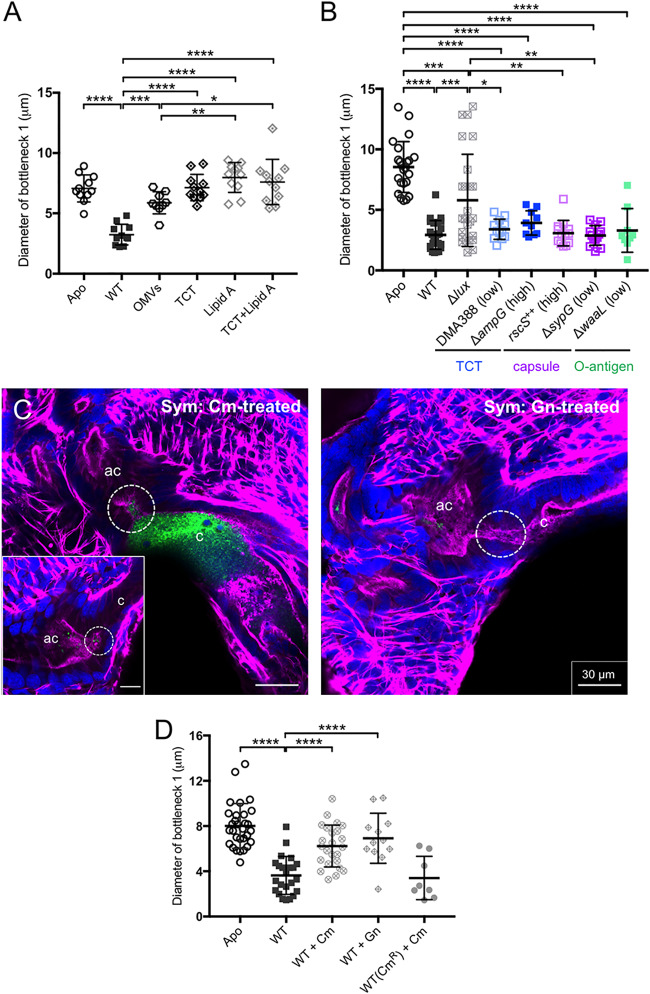
Conditions with potential effects on the bottleneck response. (A) Bacterial products and microbe-associated molecular patterns were incubated with the animal for 24 h. The following concentrations were used for each: outer membrane vesicles (OMVs), 100 μg·ml^−1^; tracheal cytotoxin (TCT), 1 μM; lipid A, 10 μg·ml^−1^. For the combination of TCT and lipid A, a combination of each concentration tested in isolation was used. A one-way ANOVA and Tukey’s *post hoc* test were used (*F*_6, 64_ = 21.59, *P* < 0.0001), For both aposymbiotic and symbiotic states, *n* = 11; for TCT and lipid A separately and for the combination of TCT and lipid A, *n* = 11; for OMVs, *n* = 9. (B) Genetic mutant strains of wild-type ES114 (WT) (Table 1) were used to assess the bottleneck diameters of 24-h symbiotic animals compared to diameters in aposymbiotic animals. Δ*lux* strain, no luminescence; DMA388 and Δ*ampG* strain, TCT production; Δ*sypG* and *rscS^++^* strains, capsule production; Δ*waaL* strain, no O antigen. A one-way ANOVA and Tukey’s *post hoc* test were used (*F*_7, 121_ = 18.03, *P* < 0.0001). Numbers of animals were as follows: for aposymbiotic state, *n* = 23; ES114, *n* = 24; Δ*lux* strain, *n* = 25; DMA388 (Δ*ltgA* Δ*ltgD* Δ*ltgY*::pDMA90), *n* = 12; DMA352 (Δ*ampG*), *n* = 10; *rscS*^++^ strain, *n* = 13; Δ*sypG* strain, *n* = 13; Δ*waaL* strain, *n* = 10. (C) Representative confocal micrographs of a single *z*-plane with merged channels to show the open symbiotic bottleneck (white, dashed circle) following treatment with each antibiotic type. Left, for Cm treatment, reopened bottlenecks (white, dashed circle) are shown both when crypts were cleared (inset) and when green fluorescent protein-labeled V. fischeri cells remained (although nonluminous). Right, Gn-treated animals were clear of bacterial cells. (D) Bottlenecks of antibiotic-treated animals compared at 48 h postinoculation. Data were analyzed using a one-way ANOVA and Tukey *post hoc* test. Numbers of animals were as follows: for aposymbiotic state, *n* = 30; wild type, *n* = 23; wild type treated with Cm, *n* = 24; wild type treated with Gn, *n* = 12; Cm^r^ wild type treated with Cm, *n* = 8 (*F*_4, 92_ = 21.78, *P* < 0.0001). Values that are significantly different are indicated as follows: *, *P < *0.05; **, *P < *0.01; ***, *P < *0.001; ****, *P < *0.0001. ac, antechamber; c, crypt; Cm, chloramphenicol; Cm^r^, chloramphenicol resistant; Gn, gentamicin. WT, wild type.

Symbiont luminescence, outer membrane vesicles (OMVs), microbe-associated molecular patterns (MAMPs, notably lipid A, O antigen, and the peptidoglycan monomer), and capsule, alone and/or in synergy, induce many developmental phenotypes of the light organ (35, 74). Here, using both V. fischeri mutants and purified active biomolecules where appropriate, none of these elements controlled the dynamics of the bottleneck to the same extent as living V. fischeri (Fig. 5A and B and Table 1; Fig. S6). The most variation was with the mutants in light production; bottleneck tissue remained open with colonization in some animals but, similar to wild-type tissue, was constricted in others. Given that the bottleneck response to mutants in light production (ES114 Δ*lux*) (Fig. 5B) was similar to that of luminous wild-type MB13B1 (Fig. 4A and B), it is unlikely that the level of light from the symbionts is the principal cue for bottleneck constriction.

**TABLE 1:** A list of the strains and plasmids used in this study

Strain or plasmid	Phenotype^*a*^	Description	Reference(s)
Strains			
ES114	Wild type (S strain)	E. scolopes light organ isolate; sharing behavior	(32, 65)
MB13B1	Wild type (S strain)	E. scolopes light organ isolate; sharing behavior	(32, 66)
MB13B2	Wild type (D strain)	E. scolopes light organ isolate; dominant behavior	(32, 66)
KB2B1	Wild type (D strain)	E. scolopes light organ isolate; dominant behavior	(32, 66)
EVS102	Nonluminous	ES114 Δ*luxCDABEG* (VF_A0923-0918); locus carrying the *lux* genes	(67)
VCW3F6	Lysine auxotrophy	ES114 *lysA*::Tn*kan* (VF_2485); lysine synthesis	(68)
*flrA::kan* strain	Nonmotile	ES114 *flrA*::*kan* (VF_1856); flagellum synthesis regulator	(69)
DMA388	Low export of TCT	ES114 Δ*ltgA* Δ*ltgD* Δ*ltgY*::pDMA90; triple transglycosylases	(70)
DMA352	High export of TCT	ES114 Δ*ampG* (VF_0720); muropeptide transporter	(70)
KV1787	Decreased capsule formation	ES114 Δ*sypG* (VF_A1026); *syp* polysaccharide synthesis activator	(71)
KV4366	Increased capsule formation	ES114; *rscS*^++^ (VF_A0237); *syp* polysaccharide synthesis activator	(64)
MB06859	No O antigen on LPS	ES114 *waaL*::Tn*erm* (VF_0151); O-antigen ligase	(72)
Plasmids			
pVSV102	GFP, Kan resistance	Used for fluorescent labeling	(73)
pVSV105	Cm resistance	Used for genetic complementation	(73)

a S, sharing; D, dominant; GFP, green fluorescent protein.

10.1128/mBio.00853-20.5 FIG S5 Antibiotic effects on symbiotic bottlenecks for animals colonized by different strains. Symbiotic bottleneck diameters varied substantially after curing with chloramphenicol (Cm) depending on the wild-type strain that colonized crypt 1. Antibiotic-treated animals were first colonized for 24 h, cured for 24 h, and then compared at 48 h. A one-way ANOVA and Tukey’s *post hoc* test were used (*F*_3, 53_ = 44.98, *P < *0.0001) (apo, *n* = 22; ES114, *n* = 12; ES114 with Cm treatment, *n* = 21; MB13B1, *n* = 20; MB13B1 with Cm treatment, *n* = 15, MB13B2, *n* = 24; MB13B2 with Cm treatment, *n* = 18). Values that are significantly different are indicated as follows: **, *P < *0.01; ***, *P < *0.001; ****, *P < *0.0001. Download FIG S5, TIF file, 14.1 MB. Copyright © 2020 Essock-Burns et al. 2020 Essock-Burns et al. This content is distributed under the terms of the Creative Commons Attribution 4.0 International license.

10.1128/mBio.00853-20.6 FIG S6 V. fischeri features that induce host light organ developmental phenotypes as candidates for inducers of bottleneck constriction. Features of V. fischeri cells tested in this study are in boldface. Components of the lipopolysaccharide (LPS) portion of the outer membrane (dashed box) contain four parts: the outermost capsule (dark green), O antigen (magenta), core polysaccharide (purple), and lipid A (bright green). Peptidoglycan (PGN) comprises the cell wall (orange), dividing the outer and inner membranes. The PGN monomer, tracheal cytotoxin (TCT), is exported and can be incorporated into the PGN layer or exported to the external environment (orange arrow). Outer membrane vesicles (OMVs) bleb from the outer membrane, around the cell body, and near the flagellar pole (60); they contain an active PGN derivative but not TCT (61). Light (blue arrow) is produced when V. fischeri cells are in high density. Download FIG S6, TIF file, 14.1 MB. Copyright © 2020 Essock-Burns et al. 2020 Essock-Burns et al. This content is distributed under the terms of the Creative Commons Attribution 4.0 International license.

To determine whether the maintenance of bottleneck closure requires the symbionts to continuously signal the host, we asked whether it remains closed after antibiotic treatment to eliminate symbionts. We exposed 24-h-colonized juvenile squid to one of two types of antibiotics, the bacteriostatic chloramphenicol (Cm) or the bactericidal gentamicin (Gn), which have previously been shown to reduce CFU abundance to undetectable levels (36). At 24 h after treatment with either antibiotic, the bottleneck 1 diameters of treated hosts were significantly larger than those of untreated, colonized animals (Fig. 5C and D). To verify that this bottleneck deconstriction was due to loss of bacteria and to control for potential off-target effects of the antibiotics, we also colonized animals with a Cm-resistant derivative of ES114 and then treated them with Cm. The bottlenecks of these animals resembled those of wild-type colonized, untreated animals (Fig. 5C and D). Treatment with Gn typically cleared ES114 cells from crypt 1, and the bottleneck always reopened under these conditions (Fig. 5C and D). On occasion, abundant, but unculturable, ES114 cells were observed in crypt 1 after Cm treatment, and the bottleneck had still opened (Fig. 5C). Taken together, the data show that continued symbiont presence was necessary for the normal bottleneck response. After antibiotic treatment, V. fischeri cells were observed more often in crypts 2 and 3; this result was expected, given that these crypts do not vent as reliably. To determine whether these remaining cells were metabolically active, we homogenized a subset of these treated animals. The cells were no longer luminous, and the numbers of viable CFU were reduced to ∼0.04% (in Cm) and ∼0.001% (in Gn) of untreated symbiotic animals (∼1.65 × 10^5^ CFU·ml^−1^).

**FIG 6:**
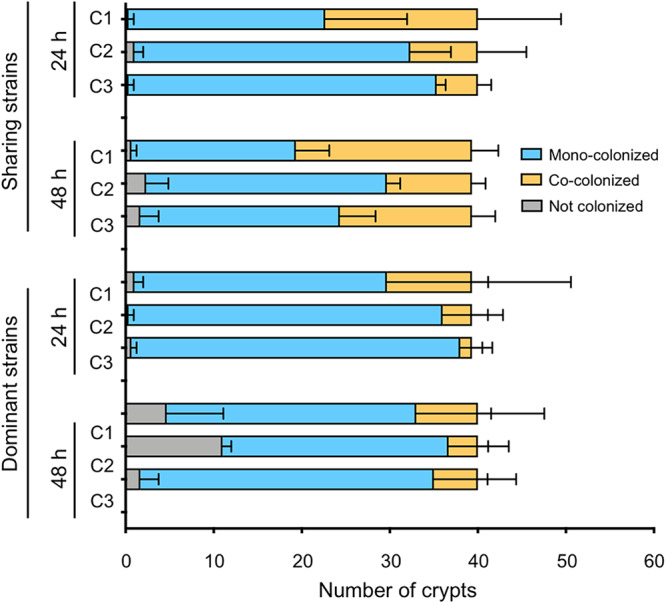
State of colonization of juvenile squid after coinoculation with a mixture of either two dominant-type strains or two sharing-type strains. Values are means of three experiments per condition. Proportion of each crypt type (C1 to C3) that was monocolonized or cocolonized (both strains) is shown, as indicated, at 24 h and 48 h postinoculation. Sharing-type strains, MB13B1 and ES114; dominant-type strains, KB2B1 and MB13B2 (Table 1).

Given that the likelihood of survival may differ among V. fischeri cells depending on the crypt they occupy (Fig. 2D), their proximity to host tissue (Fig. 2C), and the crypt’s venting behavior (Fig. 3D), we asked whether the three crypts showed differences in their propensities to support cocolonization. Specifically, we coinoculated strains characterized by either dominant or sharing behavior and looked at the prevalence of cocolonization in each crypt over the first 2 days of symbiotic development. The sharing strains (i.e., ES114 and MB13B1) cocolonized all crypts, and these cocolonizations increased substantially from 24 h to 48 h (Fig. 6). In contrast, as expected, dominant strains (i.e., KB2B1 and MB13B2) generally exhibited fewer shared crypts; however, the level of cocolonization increased from 24 to 48 h for both crypt 1 and crypt 3 (Fig. 6). These data suggest that although crypts with different states of tissue maturation at hatching (i.e., the more mature crypt 1 and the less mature crypt 3) have substantial differences in morphology, they are equally capable of successfully supporting cocolonizations over time.

Finally, we sought to determine whether the V. fischeri cells of crypt 3, which label as live after antibiotic treatment, might provide a reservoir for the subsequent recolonization of crypts 1 and 2 after the lifting of antibiotic pressure. We colonized animals for 24 h and then treated them with levels of antibiotics (Cm or Gn) that were either sustained or removed for 24 or 48 h. At 4 days postinoculation, animals that had received a 24-h pulse of Cm, followed by a 2-day recovery, were recolonized; they exhibited luminescence readings comparable to those of the 96-h symbiotic controls and had comparable CFU levels (Table S1). In addition, after 2 days of Cm relief, crypt 1 was more often colonized by live cells in the majority of animals (5/7 animals), whereas 1 day of relief was not sufficient, and live cells were rarely observed (1/8 animals) (Table S2 and Fig. S7). Due to the facts that crypt 3 retained live symbionts after 1 day of treatment with either antibiotic (14/15 animals) and that live cells were observed in the migration path to crypt 1 in all groups (Table S2), these two populations of live cells may be the source of cells recolonizing crypt 1. Thus, the data suggest that the symbiont cells in crypt 3 are under more stressful conditions. The biochemical and physiological states of those cells could be responsible for rendering them less susceptible to antibiotic treatment, possibly providing a reservoir for repopulating crypts should colonization be compromised.

10.1128/mBio.00853-20.7 FIG S7 Symbiont recovery in crypts after curing. The heat map depicts prevalence of live V. fischeri cells in each crypt (C1 to C3) after 24-h symbiotic animals were treated with antibiotics (Ab) and given relief from antibiotics (corresponds to Table S2). The number of crypts with V. fischeri present after chloramphenicol treatment (A) or gentamycin treatment (B) is shown. Relief consisted of washes with filter-sterilized seawater (FSW). Download FIG S7, TIF file, 14.1 MB. Copyright © 2020 Essock-Burns et al. 2020 Essock-Burns et al. This content is distributed under the terms of the Creative Commons Attribution 4.0 International license.

10.1128/mBio.00853-20.9 TABLE S1 Effects of antibiotic treatment and relief on symbiont luminescence and population within a host. Download Table S1, PDF file, 0.1 MB. Copyright © 2020 Essock-Burns et al. 2020 Essock-Burns et al. This content is distributed under the terms of the Creative Commons Attribution 4.0 International license.

10.1128/mBio.00853-20.10 TABLE S2 Prevalence of live symbionts within host tissues after antibiotic treatment. Download Table S2, PDF file, 0.1 MB. Copyright © 2020 Essock-Burns et al. 2020 Essock-Burns et al. This content is distributed under the terms of the Creative Commons Attribution 4.0 International license.

## DISCUSSION

An essential process during host embryogenesis of a horizontally transmitted symbiosis is the development of molecular, cellular, and anatomical features that promote the eventual engagement of and colonization by coevolved microbial partner(s) (37). Although past studies of the squid-vibrio system had outlined the embryonic and early postembryonic development of the nascent symbiotic tissues (16, 17, 26), the work presented here demonstrates that a critical event during the first ventilations that follow hatching are the opening and expansion of the region in preparation for symbiont migration into host tissues (Fig. 1A). This event triggers the creation of the migration paths to the crypts, starting at the ciliated/microvillous pores and proceeding to the ducts, antechambers, and bottlenecks. The mechanisms underlying this process remain to be determined, but are biophysically analogous to the rapid changes at birth in the volume and length of the human lung (38), which also facilitates interactions of the tissues of the respiratory pathway with the microbial world. The involvement of such precipitous events is likely only to be characteristic of horizontally transmitted symbioses that ensue shortly after birth or hatching.

**FIG 7:**
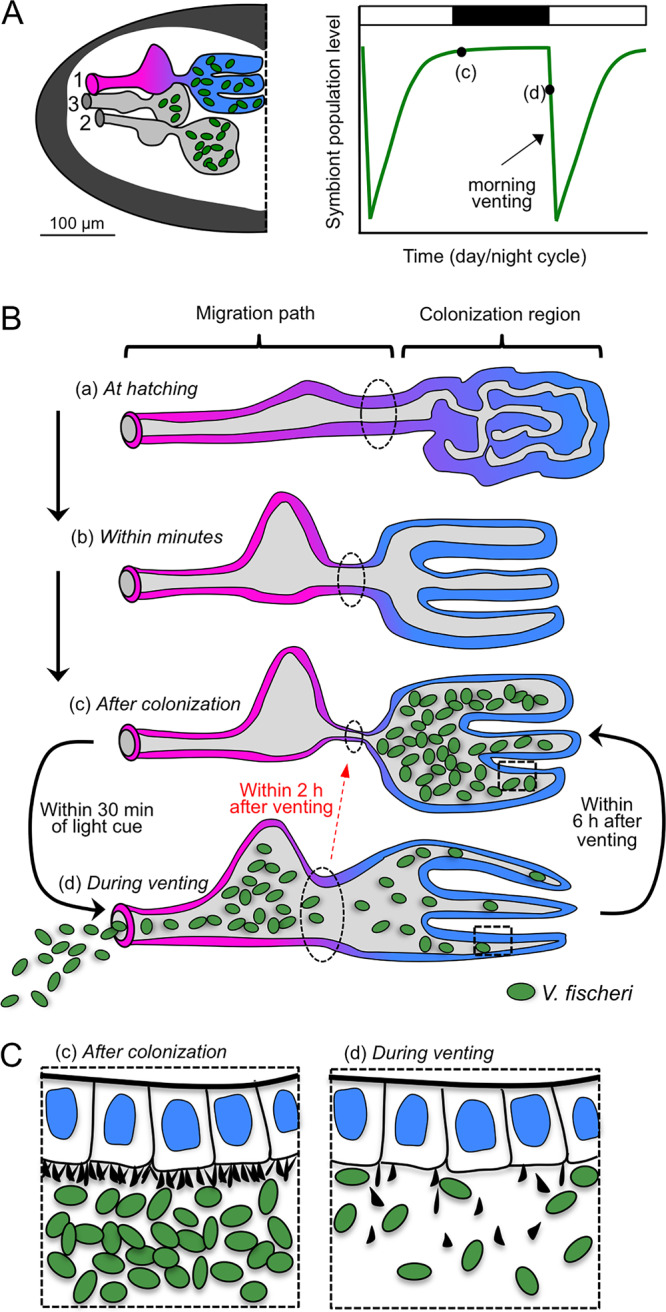
Bottleneck tissues over developmental time and symbiotic state. (A) Diagram of inner structures shown on one half of the light organ (left). The dashed line indicates the medial, and numbers indicated the migration path and crypt number, corresponding to the tissue maturity (1 > 2 > 3). The schematic at right shows symbiont growth over the day/night cycle, illustrating expulsion at dawn and outgrowth throughout the day, with the points labeled c and d corresponding to tissue labels in panels B and C. (B) Changing tissue regions of the migration path (magenta) and the site of colonization (blue). The bottleneck (black, dashed circle) constricts with symbiont colonization (c) and widens concurrent with the dawn light cue (d), when symbionts are expelled. The return of bottleneck constriction occurs within 2 h of the dawn light cue (red arrow), while the symbiont regrowth occurs over 6 h (c). After the initial symbiotic transition of the bottleneck from open (b) to closed (c), there is widening associated with venting. (C) After colonization, dense microvilli (black projections on the apical cell surfaces) form the crypt epithelial brush border (c). Microvilli are effaced during expulsion of bacteria (d).

In this study, we compare the triggering of the bottleneck’s constriction to the other known symbiosis-induced developmental events in the squid-vibrio system (35). The data provide evidence that this constriction is a defining element, being essential for both restricting initial access to the crypts and for the first outgrowth of the symbionts in the crypt spaces (Fig. 7). This behavior may also play a role in limiting subsequent colonization. Although variation among strains occurs in the timing and extent of the narrowing of the bottleneck, this feature uniformly appears to retain the symbionts in the crypt regions, except during venting when the bottleneck opens transiently (Fig. 3). However, the finding that during an MB13B1 colonization an open bottleneck can occur where symbionts are also confined to the crypts suggests that other factors may act synergistically to drive this phenotype. Further, treatment with antibiotics showed that persistent interactions with symbionts are essential for the dynamic behavior of the bottleneck.

This sort of restrictive biogeography occurs widely in binary associations, from the nitrogen-fixing symbionts of the legume root nodule to the exosymbionts on the cuticles of attine ants and the bacterial partners in the vesicles off the foregut in certain nematodes (for a review, see reference 39). One particularly strong comparison to the squid-vibrio association in a binary symbiosis is the partnership between the bean bug Riptortus pedestris and its specific bacterial partner Burkholderia insecticola (40). This symbiosis occurs as outpocketings, which are also called crypts, of the posterior midgut of the insect, and colonization events are very close in timing to that of the squid-vibrio system (41). Thus, as in the squid-vibrio symbiosis, it is possible to derive, with high resolution, the spatiotemporal dynamics of colonization of this insect association (41). Particularly relevant here is the finding that, during initial symbiont colonization of nymphs, B. insecticola cells pass through a constricted region about the same length as the ∼25-μm light organ bottleneck of juvenile E. scolopes (41). In addition to the bean bug-*Burkholderia* gut symbiosis (40, 41), recent studies on the green bug-*Pantoea* gut symbiosis also provide another intriguing analogous system that displays dynamic remodeling of constriction and crypts of the symbiotic organ through the postembryonic development of the host animals (42). In these systems, as in the squid-vibrio symbiosis, specific host-symbiont interactions facilitate a successful initiation of the symbiosis and its persistence.

Biogeographic patterns that are influenced by interactions with symbionts have also been noted in consortial gut symbioses. A recent study showed that the monogastric gut of a newborn ruminate requires interactions with microbial symbionts to differentiate into the characteristic chambered stomach with the rumen, reticulum, and omasum (43). In addition, symbiont colonization of germfree mice leads to changes in the epithelium of the gastrointestinal tract, such as the shortening of the intestine (44) and the generation of adaptive lymphoid tissues (45). On a finer scale, studies of the transcriptome in germfree and conventionalized mice revealed symbiont-induced, predictable transcriptomic responses in particular cell populations of the small intestinal and colonic villi (46). Taken together, the data demonstrate that species-specific aspects of the biogeography of colonized tissues are critical drivers of symbiosis onset throughout the evolution of animals.

Also shared across many symbioses is the induction of host development in response to cell surface molecules of the symbionts, including exopolysaccharides, lipopolysaccharide (LPS), and peptidoglycan (PGN) derivatives, as well as flagella (35, 45, 47, 48). Because LPS and PGN derivatives, as well as light (48), are so active in driving development in the squid-vibrio system, we both genetically manipulated these elements in the symbionts and used pharmacological treatment (e.g., addition of purified MAMPs) to determine whether bottleneck constriction was also controlled by these factors; however, we found none of them to be sufficient to drive bottleneck closure. Thus, future studies will be directed toward determination of the active effectors responsible for this phenotype.

An important feature in host-symbiont interactions is the extent of development of host tissues. The tissue maturation state is a key factor in early interactions with the developing microbiota in mammals (49–51). For example, the gut microbiome of premature infants, in the weeks to months following birth, differs from that of infants with a full-term gestational period (52); these infants face risks of developing necrotizing enterocolitis, an intestinal disease with links between the immature tissue state, the immune system, and exposure to microbes (52–55). While differences in maturation have been noted in these pathologies, the authors are not aware of studies that have defined whether such variation in the maturation of microenvironments occurs along the newborn mammalian gut under healthy conditions.

The level of crypt maturation naturally varies among hatchling squid, offering the opportunity to determine the impact of such differences on the process of colonization. Previous studies had identified differences in crypt sizes, which correlated directly with symbiont population levels in the colonized crypt spaces (16). In addition, variation in daily expulsion behavior had been documented, with the most mature hatchling crypt (crypt 1) expelling most of its symbionts at the first dawn venting and the least mature (crypt 3) not expelling any colonizing V. fischeri at this time (17). The data here have greatly expanded our view of maturation differences among the crypts. The crypts, from most mature to least mature (i.e., from crypt 1 to 3), range in features including, respectively: (i) from intimate contact to limited contact between partner cells along the crypt epithelia, (ii) from either few to no dead cells, to many dead cells in the crypt spaces, and (iii) from low to high resistance of the crypt symbionts to antibiotic treatment. While the mechanisms underlying these differences will require future study, this variation has implications for both partners. The lack of initial venting behavior of crypt 3 and its tendency to support symbionts in an environment more resistant to perturbation (reduced metabolism, decreased nutrients, and less exchange with the host) present the intriguing possibility that this crypt acts as a reservoir, or insurance policy, should the initial colonization of the hatchling’s more mature crypts be lost. In contrast, the lack of initial venting may well allow cheaters (i.e., dim or dark symbionts) to persist in crypt 3; while these light-defective strains are eliminated from crypt 1 within the first few days of colonization, they persist in crypts 2 and 3 for several weeks (56, 57). Despite these differences in maturation states of the crypts at hatching, the three crypts on either side of the organ can be colonized by symbionts and, eventually, with continuing postembryonic development, become anatomically indistinguishable (58). Thus, this trajectory suggests the presence of evolutionary selection on the initial posthatch variation in maturation state of the crypts.

The data presented here demonstrate that features in both host and symbiont, notably variation in host tissue maturation and symbiont strain variation, are strategies ensuring the successful colonization and persistence of the squid-vibrio association. With its high spatiotemporal resolution, these data provide a rare window into the dynamic physical landscape that creates a stable symbiosis. In addition, these findings open the door for a vast array of future investigations, including the determination of the bacterial element responsible for the bottleneck phenotype, the biochemical barriers that prevent subsequent colonization, and the spatiotemporal host responses mediating bottleneck dynamics. The ultimate goal of these studies is to provide insights into how the events occurring during initial colonization drive the establishment of a healthy partnership in both simple and complex symbioses.

## MATERIALS AND METHODS

### General.

Unless otherwise noted, all chemicals were purchased from Sigma-Aldrich (St. Louis, MO), and fluorescent dyes were from Thermo Fisher Scientific (Waltham, MA). Adult E. scolopes animals were collected from Maunalua Bay, Oahu, and maintained as previously described (16). Juvenile squid were transferred into synthetic seawater (Instant Ocean [IO]; Aquarium Systems, Mentor, OH) within 5 min of hatching to examine hatchling tissue morphology. All other experiments were done in natural offshore seawater, either unfiltered or filtered (FSW; 0.22-μm pores size), which produced no significant difference in results. Individual hatchlings were inoculated with V. fischeri cells at a concentration of 5 × 10^3^ to 1 × 10^4^ CFU·ml^−1^ in seawater, and luminescence was monitored with a TD-20/20 luminometer (Turner Designs, Inc., Sunnyvale, CA) as a measure of a successful colonization (59). To cure colonized animals, animals were placed in FSW containing 20 μg·ml^−1^ chloramphenicol (Cm) (36) or 200 μg·ml^−1^ gentamicin (Gn) for 24 h. Estimates of symbiont population levels in colonized light organs were determined by counting CFU on Luria-Bertani salt (LBS) medium (10 g of Bacto tryptone, 5 g of yeast extract, 20 g of NaCl, and 50 ml of 1 M Tris-HCl buffer [pH 7.5] per liter of deionized water) plates (60).

### Bacterial products.

Outer membrane vesicles (OMVs) produced by V. fischeri ES114 were prepared as previously described (61, 62), with the following modification. After OMVs were separated from other extracellular products by ultracentrifugation, they were further purified by using linear 10 to 50% sucrose gradient centrifugation at 100 000 × *g* for 16 h at 4°C in a 90 Ti rotor (Beckman Coulter, Inc., Brea, CA). To treat squid with OMVs, 100 μg·ml^−1^ was added to FSW and incubated with hatchling animals for 24 h (61, 62). The peptidoglycan monomer (tracheal cytotoxin [TCT]) and lipid A from V. fischeri were added to FSW for 24 h at a concentration of 1 μM and 10 μg·ml^−1^, respectively (25, 26).

### Sample preparation and microscopy.

Juvenile squid were transferred to 4% paraformaldehyde in marine phosphate-buffered saline (mPBS; 50 mM sodium phosphate buffer, 450 mM NaCl, pH 7.4) and fixed overnight at 4°C and then washed three times for 30 min in mPBS prior to removal of the light organ by dissection. Light organs were permeabilized and stained in 0.1% Triton X-100 in mPBS for 1 to 2 days in the dark at 4°C. Once excess dye was washed off, samples were mounted in Vectashield (Vector Laboratories, Burlingame, CA) and overlaid with a coverslip (number 1.5, Fisherbrand; Fisher Scientific, Waltham, MA) in such a way as to limit the *z* dimension of the light organ prior to imaging. A 1:40 dilution of phalloidin in mPBS (with either rhodamine, with excitation/emission [Ex/Em] at 540/565 nm, conjugated to Alexa 405, with Ex/Em at 405/450 nm) was used to stain F-actin. Nuclei were visualized using TOPRO-3 (Ex/Em, 642/661 nm; 1:1,000 dilution). A fixable stain for dead cells was incubated with the animal for 6 h prior to fixation, using Live-or-Dye NucFix Red (Ex/Em, 520/593 nm) (Biotium, Fremont, CA). The dye powder was dissolved in 50 μl of dimethyl sulfoxide (DMSO) and then diluted 1:2,000 in FSW to stain the squid.

The majority of the laser scanning confocal microscopy was performed using an upright Zeiss LSM 710 confocal microscope (Carl Zeiss AG, Jena, Germany), located at the University of Hawai‘i, Mānoa (UHM), Kewalo Marine Laboratory. For image analysis, Fiji (ImageJ) was used for measurements and generation of projections of stacks (63). To increase resolution of certain structures, imaging was done on a Leica SP8 X confocal microscope (Leica Camera AG, Wetzlar, Germany), using Lightning adaptive deconvolution and Leica LasX software at the Biological Electron Microscopy Facility (BEMF) at UHM.

Samples for transmission electron microscopy (TEM) were fixed in 2% glutaraldehyde and 2% paraformaldehyde in mPBS and prepared as previously described (22, 64). Sectioning and imaging were done at BEMF, and samples were viewed on a Hitatchi HT7700 TEM at 100 kV.

### Immunocytochemistry.

A monoclonal anti-acetylated tubulin antibody produced in mouse was used to label cilia by immunocytochemistry. All incubation steps were done at 4°C on a rotator. After permeabilization in 0.5% Triton X-100 in mPBS for 2 days, samples were transferred to a blocking solution (0.5% Triton X-100, 0.5% bovine serum albumin, and 1% goat serum in mPBS) overnight. Samples were then incubated for 7 days in the primary antibody; a 1-mg·ml^−1^ stock was diluted to 1:500 in the blocking solution. Following incubation and washing in 0.5% Triton X-100 in mPBS, samples were incubated for 2 days in the secondary antibody, goat anti-mouse conjugated to tetramethyl rhodamine isocyanate (TRITC), for which a 1.5-mg·ml^−1^ stock in mPBS was diluted 1:25 in blocking solution. Following the removal of unreacted secondary antibody by washing in mPBS, samples were counterstained as described above.

### Statistical analyses.

Data were analyzed using GraphPad Prism software, version 7.0 (GraphPad Software, Inc., La Jolla, CA) and were first tested for normality using a D’Agostino-Pearson (omnibus K2) normality test. If the data passed this criterion (*P* > 0.05), then parametric tests were used, including an unpaired *t* test or a one-way analysis of variance (ANOVA), followed by Tukey’s pairwise comparison (α = 0.05). If data did deviate from a normal distribution, nonparametric tests were used, including a Kruskal-Wallis test followed by Dunn’s multiple-comparison test. To calculate the 95% confidence intervals of the proportion, the z* value used was 1.96.
