# Supplementary figures and images for: Interactions of Symbiotic Partners Drive the Development of a Complex Biogeography in the Squid-Vibrio Symbiosis
Source: mBio. 2020 May 26;11(3):e00853-20. doi: 10.1128/mBio.00853-20 (PMC7251207; doi:10.1128/mBio.00853-20)

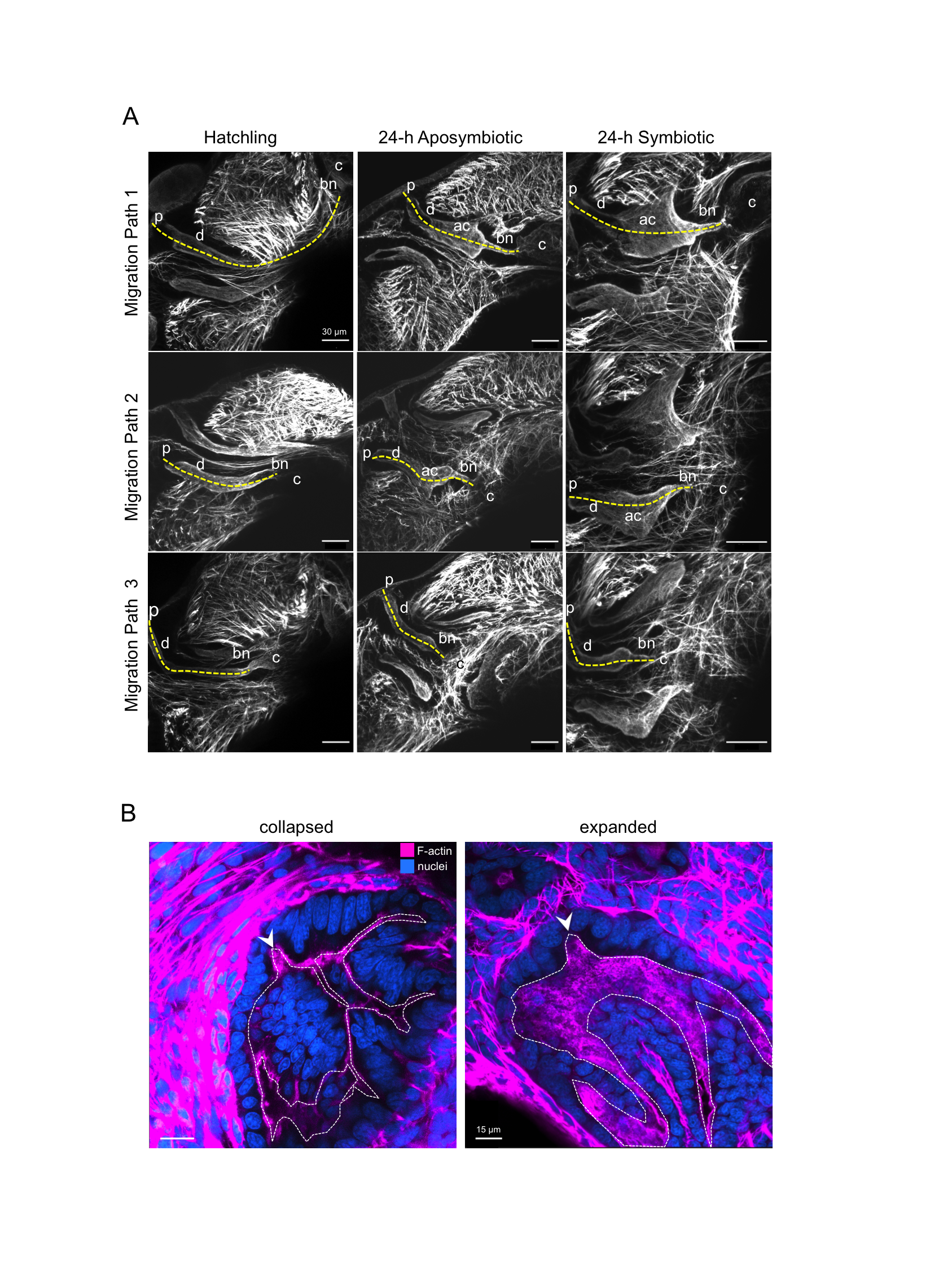

Supplement: FIG S1 [file mBio.00853-20-sf001.tif]

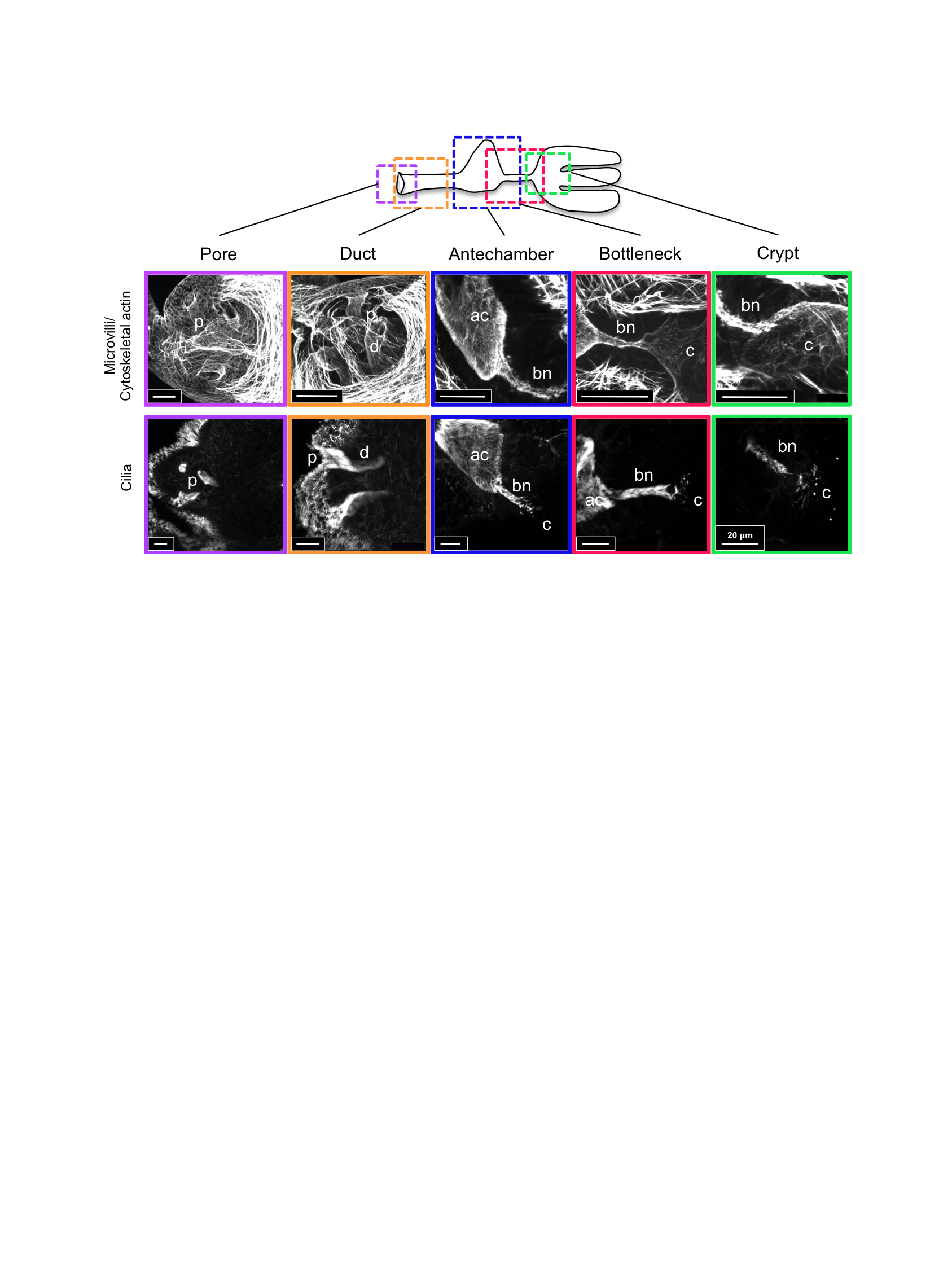

Supplement: FIG S2 [file mBio.00853-20-sf002.tif]

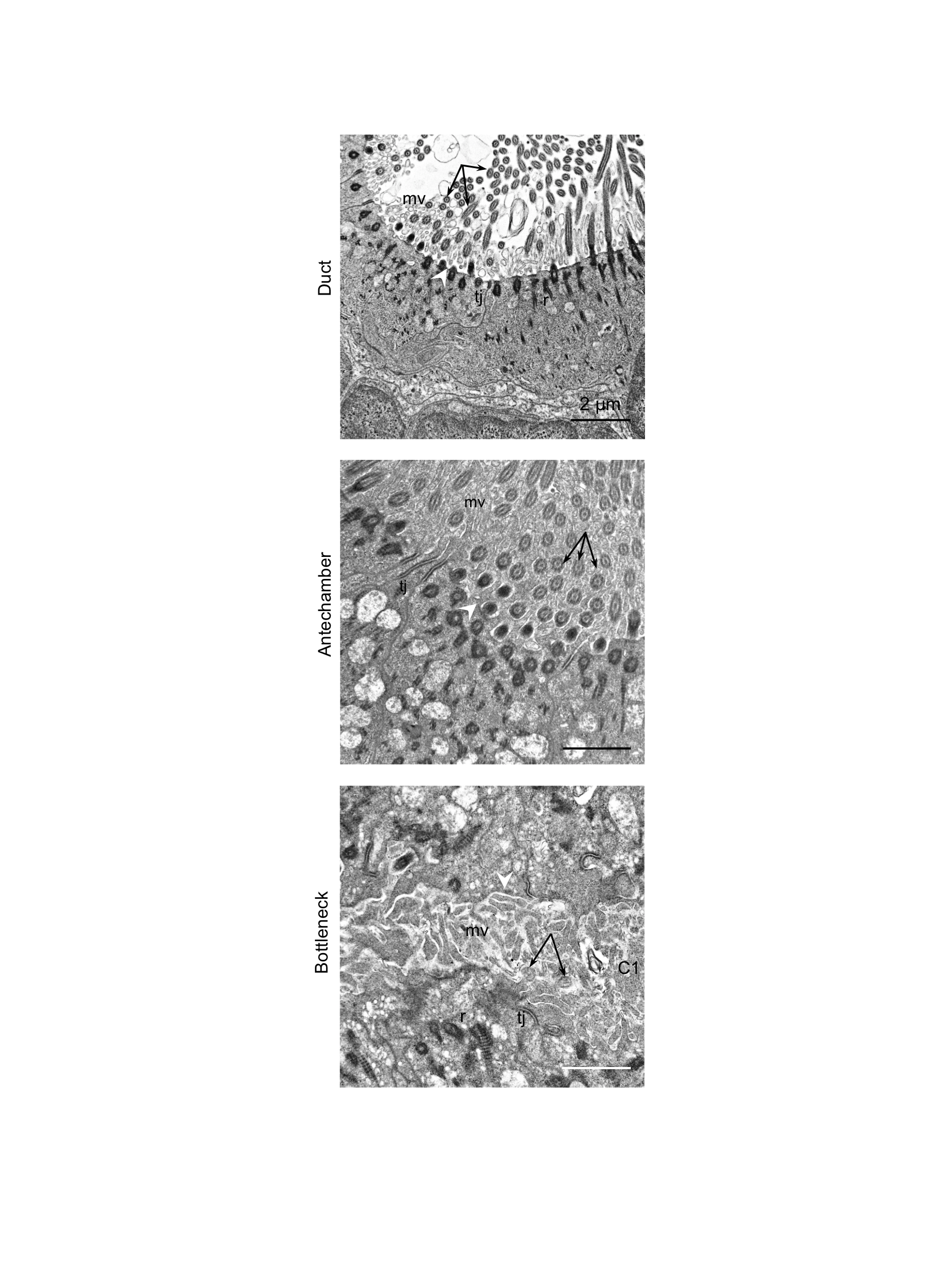

Supplement: FIG S3 [file mBio.00853-20-sf003.tif]

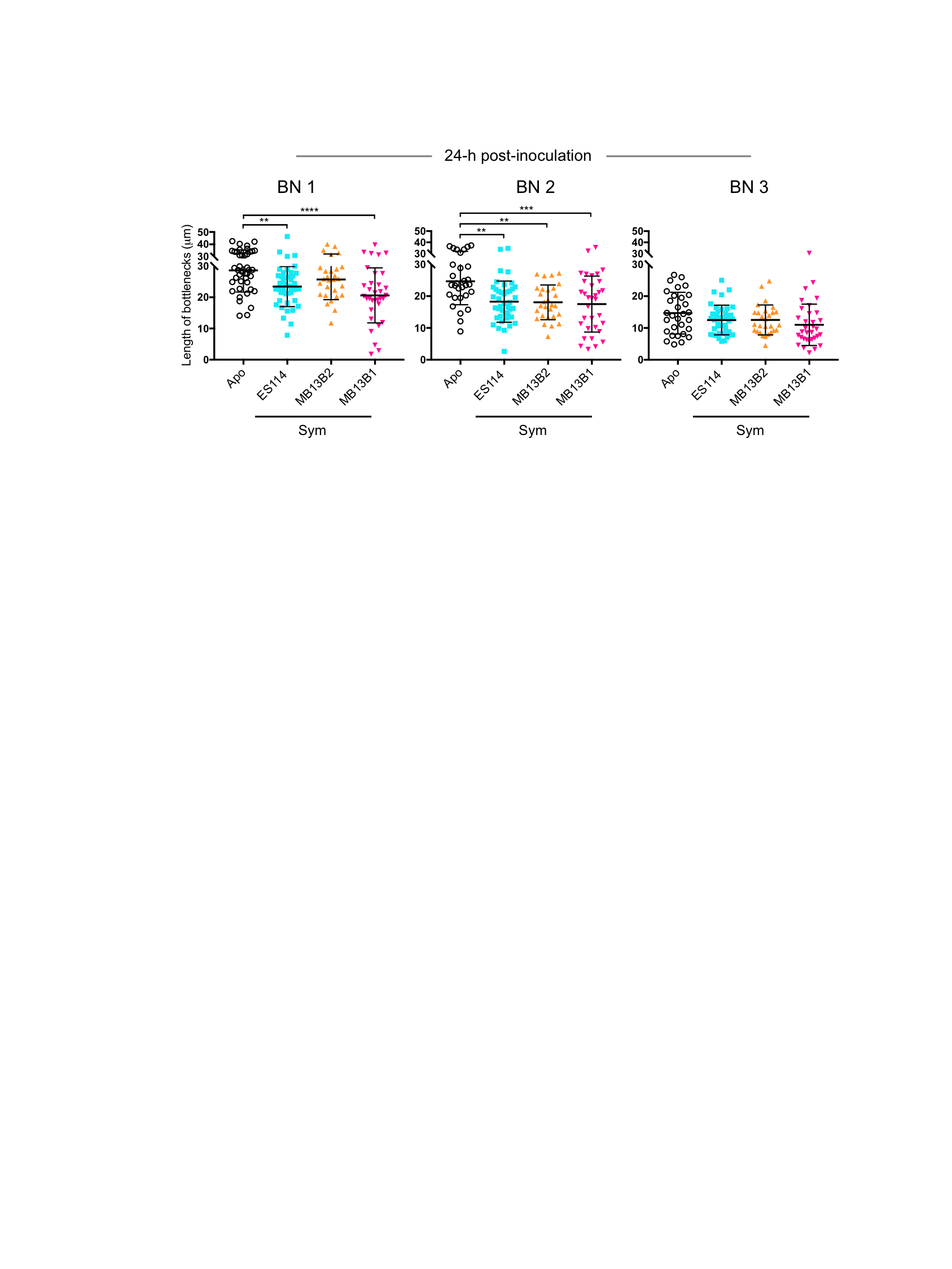

Supplement: FIG S4 [file mBio.00853-20-sf004.tif]

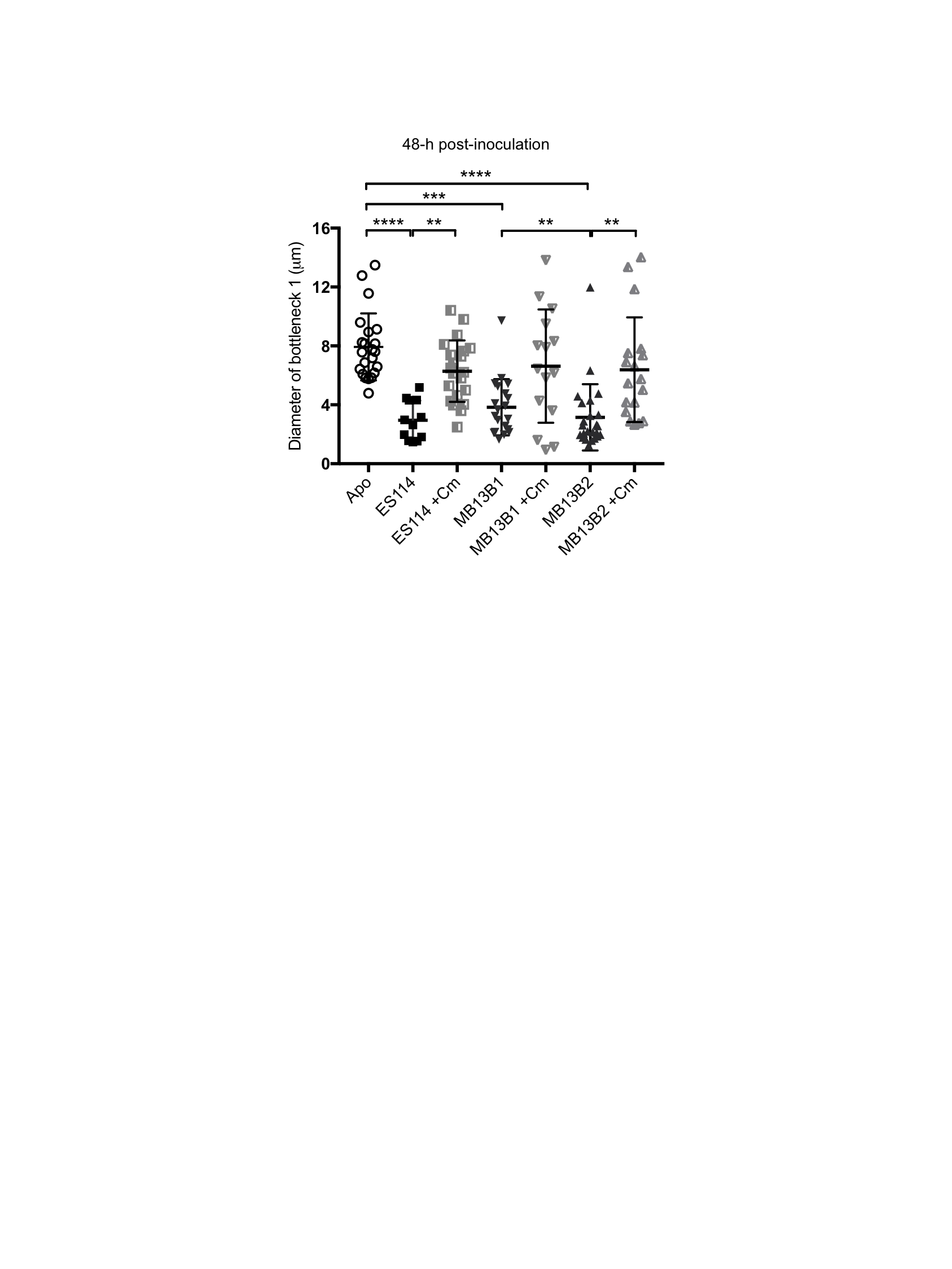

Supplement: FIG S5 [file mBio.00853-20-sf005.tif]

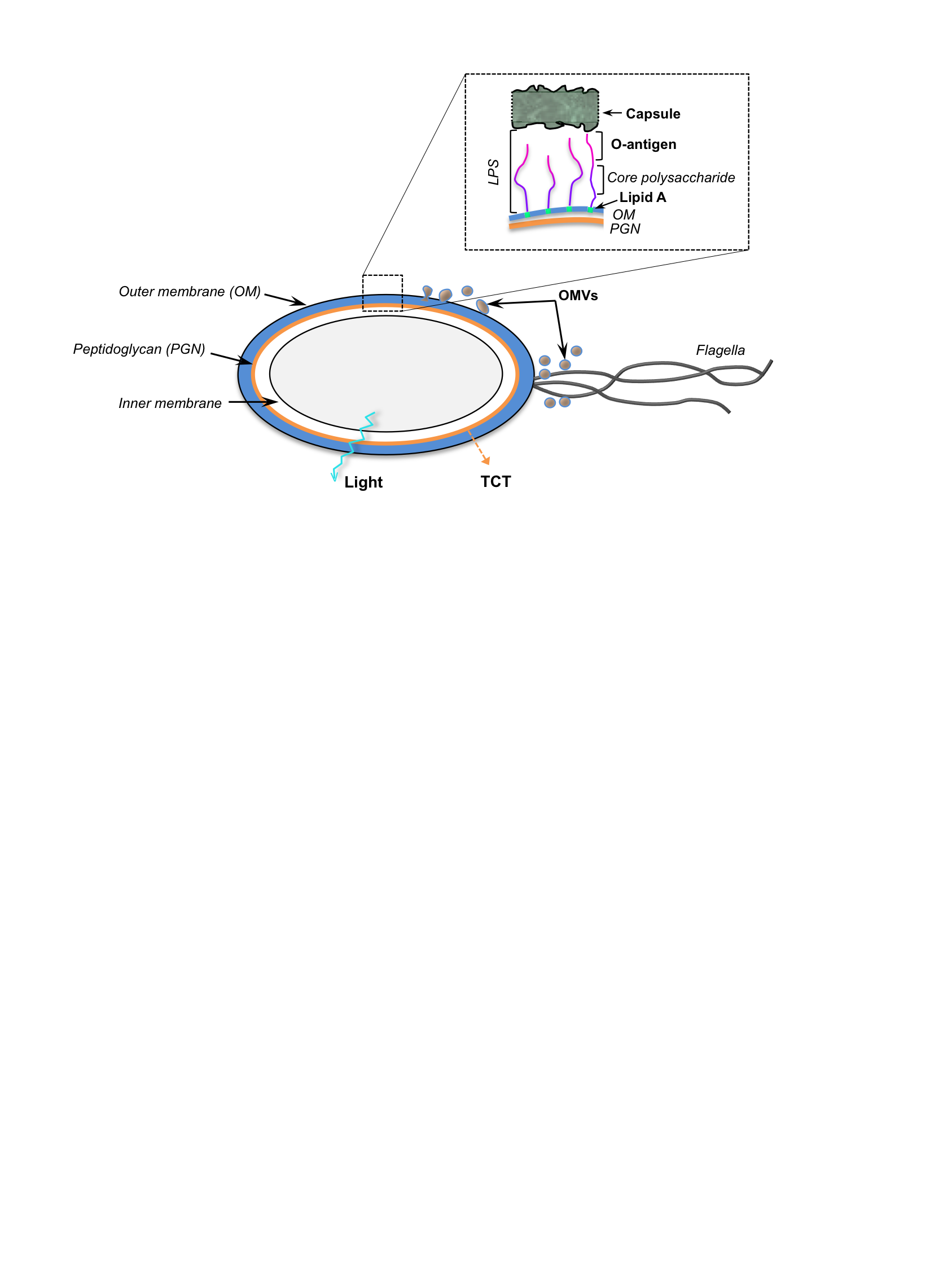

Supplement: FIG S6 [file mBio.00853-20-sf006.tif]

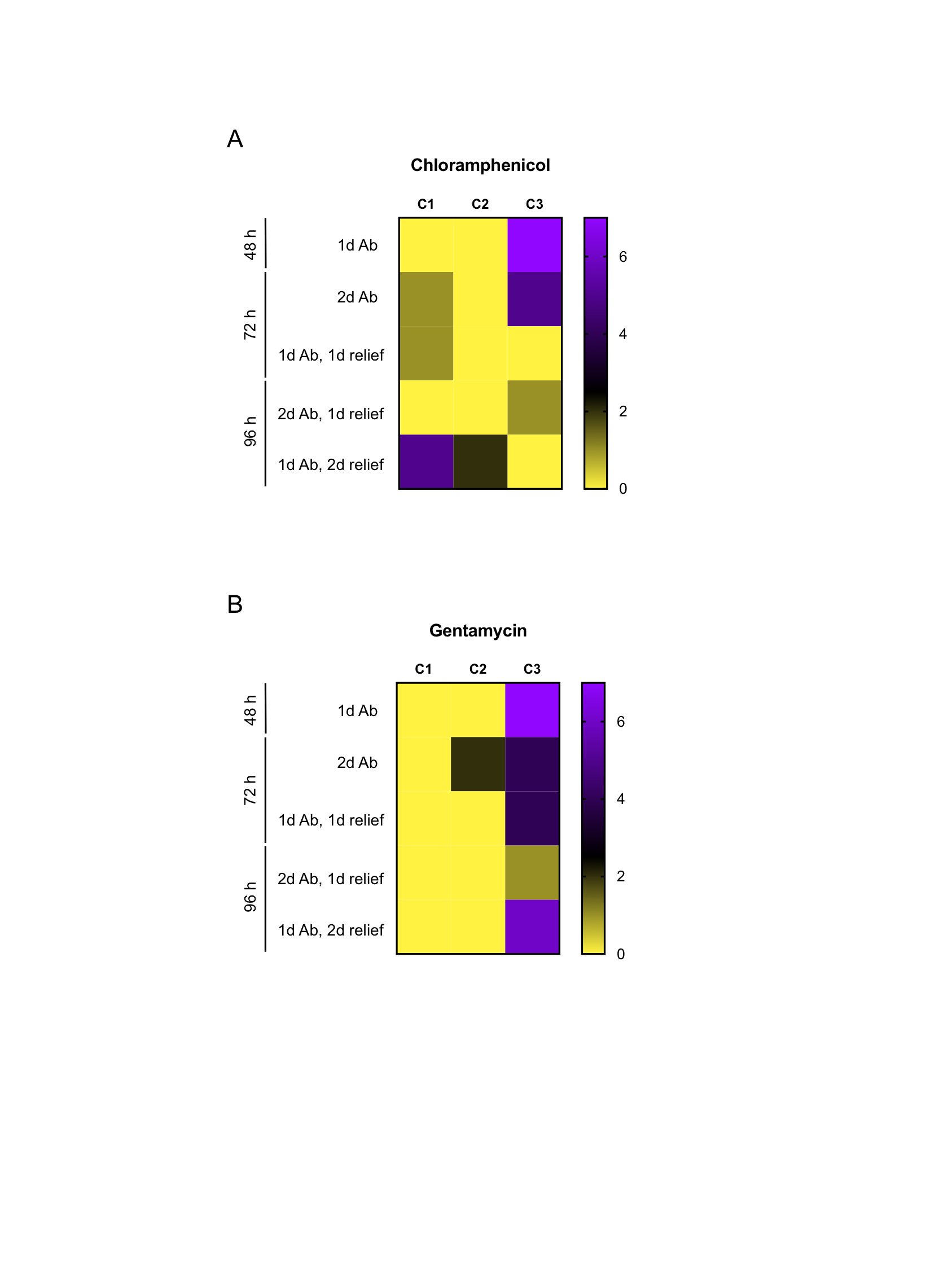

Supplement: FIG S7 [file mBio.00853-20-sf007.tif]
