# Supplementary material for: Interactions of Symbiotic Partners Drive the Development of a Complex Biogeography in the Squid-Vibrio Symbiosis
Source: mBio. 2020 May 26;11(3):e00853-20. doi: 10.1128/mBio.00853-20 (PMC7251207; doi:10.1128/mBio.00853-20)
Supplement: TABLE S1 [file mBio.00853-20-st001.pdf]

SI Table 1. Effects of antibiotic treatment and relief on symbiont luminescence and population within a host.

Treatment[a]	N	CFU/light organ[b]	Lum (RLU)[c]
24 h sym	4	8.4×10^4	487.5
48 h sym	3	1.9×10^5	454.7
72 h sym	2	5.2×10^4	31.2
96 h sym	2	2.1×10^4	17.7
48 h sym 24 h Cm	3	0	<1
48 h sym 24 h Gn	3	0	<1
72 h sym 48 h Cm	2	0	<1
72 h sym 48 h Gn	2	0	<1
72 h sym 24 h Cm	5	0	<1
72 h sym 24 h Gn	4	0	<1
96 h sym 24 h Cm	4	9.5×10^3	29.3
96 h sym 24 h Gn	4	0	<1

[a]Time post-inoculation with 5,000 cells ml *V. fischeri* – GFP, Cm is chloramphenicol; Gn is gentamycin.
[b]Average colony forming units estimated from the homogenized light organ.
[c]Average luminescence of the symbionts within the squid given as relative light units.
