# Supplementary material for: Interactions of Symbiotic Partners Drive the Development of a Complex Biogeography in the Squid-Vibrio Symbiosis
Source: mBio. 2020 May 26;11(3):e00853-20. doi: 10.1128/mBio.00853-20 (PMC7251207; doi:10.1128/mBio.00853-20)
Supplement: TABLE S2 [file mBio.00853-20-st002.pdf]

SI Table 2. Prevalence of live symbionts within host tissues after antibiotic treatment.

**Post-inoculation conditions**				**Presence of live cells**						
24 h Ab[a]	48 h Ab	72 h Ab	96 h	MP1 (max # cells)[b]	MP2 (max # cells)	MP3 (max # cells)	C1[c]	C2	C3	**N**
Cm	End			5 (*)	3 (100)	7 (15)	0	0	7	**7**
Gn	End			8 (300)	6 (12)	8 (75)	0	0	7	**8**
Cm	Cm	End		8 (200)	6 (31)	8 (220)	1	0	5	**9**
Gn	Gn	End		6 (200)	5 (36)	9 (160)	0	2	4	**9**
Cm	None	End		6 (14)	4 (7)	6 (200)	1	0	0	**8**
Gn	None	End		5 (39)	5 (23)	7 (100)	0	0	4	**8**
Cm	Cm	None	End	4 (7)	1 (15)	3 (40)	0	0	1	**6**
Gn	Gn	None	End	3 (150)	2 (60)	5 (150)	0	0	1	**6**
Cm	None	None	End	3 (18)	1 (14)	3 (65)	5	2	0	**7**
Gn	None	None	End	2 (19)	2 (20)	9 (80)	0	0	6	**9**

[a]All groups were 24-h symbiotic prior to treatment with either chloramphenicol (Cm) or gentamycin (Gn).
[b]Number of GFP-labeled *V. fischeri* present in each migration path (MP). * indicates too many to count.
[c]Number of each crypt type (C1-C3) containing >5 live symbionts.
